# Pott's Disease with Incidentally Discovered Multiple Brain Tuberculomas in a Previously Healthy 10-Year-Old Girl

**DOI:** 10.1155/2021/5552351

**Published:** 2021-04-20

**Authors:** May Albarrak, Abdulrahaman Alodayani, Nasir Al Otaibi, Yasser Albrikeet

**Affiliations:** ^1^Pediatric Infectious Diseases Section, Prince Sultan Military Medical City, Riyadh, Saudi Arabia; ^2^Spinal Surgery Department, Prince Sultan Military Medical City, Riyadh, Saudi Arabia

## Abstract

Pott's disease (PD) represents the most common form of spinal tuberculosis. Its association with brain tuberculomas is extremely rare. Herein, we report a previously healthy child with PD and concurrent multiple brain tuberculomas who was successfully treated with antituberculous therapy, surgical drainage of the paravertebral abscess, and adjuvant steroid therapy.

## 1. Introduction

Tuberculosis (TB) remains an endemic disease in the Kingdom of Saudi Arabia despite the government's efforts to eradicate the disease. According to the World Health Organization (WHO), in 2017, Saudi Arabia reported an annual TB incidence rate of 10/100,000 population and remains a moderate TB-burden country [1]. Saudi Arabia showed a stable trend of extrapulmonary tuberculosis (EPTB) over the last 5 years, 28.4% in 2012 compared to 26% in 2017. Spinal tuberculosis is a common manifestation of extrapulmonary tuberculosis (EPTB) [1, 2]. There are two main different patterns of spinal tuberculosis. The classic one is osseous spinal tuberculosis, also known as tuberculous spondylodiscitis or Pott's disease (PD), which consists of a combination of osteomyelitis of the spinal vertebrae and intervertebral discitis caused by mycobacterial tuberculosis [3]. The second uncommon pattern is nonosseous spinal tuberculosis which can manifest in several forms, such as tuberculous radiculomyelitis, syringomyelia, spinal cord tuberculoma, and rarely, spinal tuberculous abscess [4]. Brain tuberculoma is another manifestation of EPTB in the central nervous system (CNS); however, it is less frequent in comparison to meningitis. Involvement of the spinal cord or spinal nerve root is a common manifestation in patients with tuberculosis meningitis [4]. Moreover, there are some reports of cases of nonosseous spinal tuberculosis with brain tuberculoma [5]; however, the association of PD with brain tuberculoma is an extremely rare presentation [4, 6, 7]. This case report presents a rare case of PD affecting the thoracic vertebrae with concurrent multiple brain tuberculomas that were incidentally discovered by brain MRI in a previously healthy girl.

## 2. Case Report

**Figure 1:**
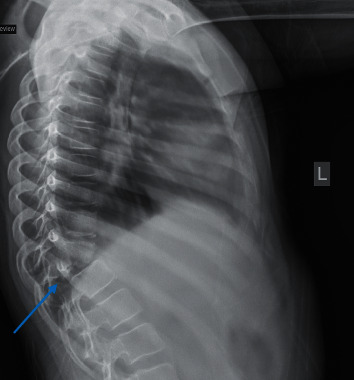
Lateral spine X-ray demonstrates destruction of the thoracic vertebra (T11) body and inferior end plate of the T10 vertebra with loss of disk spaces between T10, T11, and T12.

**Figure 2:**
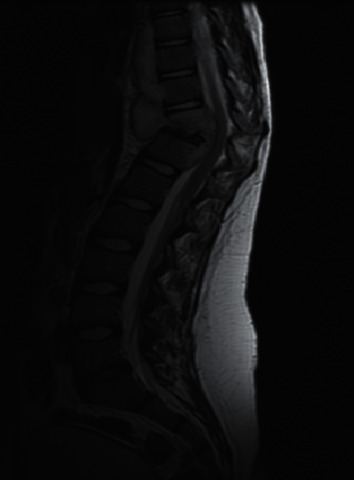
Contrasted magnetic resonance imaging (MRI) of the spine shows kyphotic destruction of thoracic vertebrae T10, T11, and T12 with abnormal high signal on T2 and low signal on T1 with heterogeneous enhancement in addition to a large heterogeneous paravertebral multiloculated prevertebral and paraspinal abscess extending from T7 through T12 with a peripheral thick wall that has low T2 and high T1 signal with intense enhancement. These are associated with an epidural collection and retropulsed collapsed T11, which result in complete attenuation of the anterior subarachnoid spaces and compression of the distal cord.

**Figure 3:**
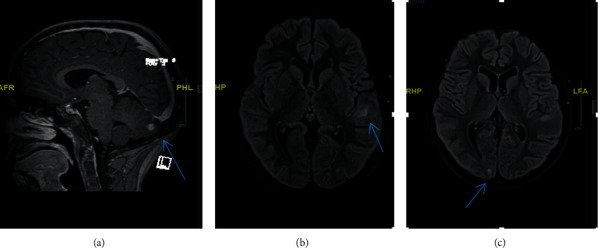
Transverse and sagittal magnetic resonance T1 contrast images of the brain show multiple tuberculomas; each appears as a ring-enhancing lesion with a hypointense center surrounded by a hyperintense area (edema). One lesion is at the left cerebellar hemisphere (a). Other small foci are at the left temporal gyrus (b) and right occipital lobe (c).

A previously healthy 10-year-old girl presented to our emergency department with a history of back pain, fever, and night sweats for 4-month duration associated with progressive walking difficulty and back deformity over the last 2 months. Physical examination revealed middle back swelling with kyphotic (gibbus) deformity. Neurological examination revealed grade 4/5 motor weakness in both lower extremities with normal deep tendon reflexes and sensations. There was no evidence of cranial nerve involvement, meningeal irritation, or cerebellar dysfunction. Laboratory tests showed a normal complete blood count, elevated C-reactive protein of 81 mg/dl, and erythrocyte sedimentation rate of 111 mm/h. Serology testing for HIV was negative. Her Mantoux test was positive of 25 mm although there was no history suggestive of tuberculosis in the family. Chest X-ray was normal; however, spine X-ray demonstrated destruction of the thoracic vertebra (T11) body and inferior end plate of the T10 vertebra with loss of disk spaces between T10, T11, and T12 (Figure 1). The contrasted magnetic resonance imaging (MRI) of her spine showed kyphotic destruction of thoracic vertebrae T10, T11, and T12 with abnormal high signal on T2 and low signal on T1 in addition to a large heterogeneous multiloculated prevertabral and paraspinal abscess extending from T7 through T12 with a peripheral thick wall that had low T2 and high T1 signal with intense enhancement. These are associated with an epidural collection and retropulsed collapsed T11, which result in complete attenuation of the anterior subarachnoid spaces and compression of the distal cord (Figure 2). She underwent open surgical drainage of the paravertebral abscess and posterior spine fixation from T6 to L2 with pedicle screws. During surgery, a massive purulent fluid was discharged, and biopsy from the bone and soft tissue was taken and sent for microbiological examination and cultures including *Mycobacterium tuberculosis*. Both acid-fast staining and mycobacterial polymerase chain reaction were negative. Histopathology examination revealed caseating granulomatous inflammation consistent with tuberculosis. Based on radiological and histopathological findings, Pott's disease was highly suspected; therefore, the decision was made to start the patient on four anti-TB drug therapy of isoniazid, rifampicin, pyrazinamide, and ethambutol) while waiting for the final result of the mycobacterial culture. Ophthalmology team was consulted for baseline eye examination before starting ethambutol, and incidentally, bilateral papilledema was detected during fundus examination. Consequently, MRI of the brain showed multiple brain lesions in the left cerebellar hemisphere, left superior temporal gyrus, and right occipital lobe (Figure 3). The lesions show a ring enhancement with a hypointense center mildly surrounded by a hyperintense area, most likely represent vasogenic edema which is highly suggestive of brain tuberculomas. There was no leptomeningeal enhancement suggestive of meningitis. After four weeks, her tissue culture grew *Mycobacterium tuberculosis*. The patient was treated with four antituberculous drug regimen for 2-month duration, followed by additional 10 months of isoniazid and rifampicin. Oral prednisone was given initially at a dose of 2 mg/kg/day for 4 weeks and tapped gradually over the next 6 weeks. In a three-year follow-up, the patient had normal neurological examination, and her brain MRI showed complete resolution of the tuberculomas.

## 3. Discussion

Both PD and brain tuberculoma usually occur from a hematogenous spread of *Mycobacterium tuberculosis* from a primary focus of infection which is usually the lungs. Nevertheless, in many cases, the primary focus cannot be detected [8]. PD in children commonly affects thoracic and lumbar vertebrae [8]. It is typically characterized by bone destruction, vertebral collapse, and kyphosis which can lead to cord compression and paraplegia. Back pain was considered as the most common presenting symptom, while other constitutional symptoms such as fever or weight loss may be absent [8]. Affected children are at risk of severe vertebral destruction and complications because of the cartilaginous nature of their bone as well as late diagnosis secondary to nonspecific clinical symptoms and the slowly progressive course of the disease [9].

Moreover, clinical presentation of intracranial tuberculomas in children is variable and mainly depends on their size and location in the brain. They can be asymptomatic in some children; however, they are typically present with seizure or symptoms of raised intracranial pressure [10].

The association of PD with intracranial tuberculomas is extremely rare. Up to date, there are only few cases which have been reported in the literature [3, 6, 7] until now, most of which were adult patients with clinical symptoms suggestive of CNS involvement. In contrast, our patient is a child who was presented only with a clinical manifestation of PD without any clinical symptoms suggestive of brain involvement; only during her baseline eye examination, a bilateral papilledema was incidentally discovered; therefore, the physician requested brain imaging to rule out the possibility of brain involvement. Her brain MRI showed multiple cranial and cerebellar ring-enhanced lesions surrounded by edema highly suggestive of brain tuberculomas with no meningeal enhancement to suspect meningitis (Figure 3).

The characteristic radiological findings of brain tuberculomas on MRI in addition to a positive Mantoux test or interferon gamma release assay or an evidence of tuberculosis in another body site are usually enough for diagnosis of brain tuberculomas, especially in area endemic for tuberculosis without the need for invasive biopsy [11]. Our patient was initially diagnosed to have PD based on the typical radiological and histopathological findings in addition to positive Mantoux test. Although both initial acid-fast smear and mycobacterial PCR were negative, the patient was empirically started on antituberculous drugs while waiting for the final mycobacterial culture result, which subsequently grew *Mycobacterium tuberculosis*, to confirm our diagnosis of PD of the thoracic spines with concurrent multiple brain tuberculomas.

Early diagnosis and treatment with antituberculous therapy are the cornerstone in the management of patients with PD and CNS tuberculosis in order to prevent associated serious complications. Surgical treatment is indicated mainly in selected patients such as patients with large abscess formation, kyphotic deformity, and evidence of spinal cord compression or failure of antituberculous therapy [12]. Adjuvant corticosteroid therapy in patients with brain tuberculomas without meningitis or spinal cord affection remains controversial although it has been used in some patients with good outcomes [13]. Our patient was successfully treated with prolonged antituberculous therapy for a total of one-year duration with adjuvant surgical drainage of the large paraspinal abscess along with oral prednisone therapy for a total of 8-week duration.

This case report emphasizes the importance of considering brain imaging in patients with PD with or without spinal cord involvement, even in the absence of clinical symptoms suggestive of brain affection. This will help to establish an early diagnosis and to start an appropriate antituberculous therapy in order to prevent the associated serious complications.

## 4. Conclusion

Association of different patterns of spinal tuberculosis with brain tuberculoma is not a very rare manifestation of EPTB [4–7]. CNS tuberculosis is a serious and often fatal disease, especially in children. Appropriate brain imaging should be considered in patients with PD even in the absence of clinical symptoms suggestive of brain involvement in order to start an early diagnosis and start a prompt anti-tuberculous management.

## Data Availability

The authors confirm that all the data previously used in the study can be found in the references and from the corresponding author upon request.
